# Stilbene Glycosides in *Pinus cembra* L. Bark: Isolation, Characterization, and Assessment of Antioxidant Potential and Antitumor Activity on HeLa Cells

**DOI:** 10.3390/plants14101459

**Published:** 2025-05-14

**Authors:** Cristina Lungu, Cosmin-Teodor Mihai, Gabriela Vochita, Daniela Gherghel, Ionel I. Mangalagiu, Mihaela Gafton, Sorin-Dan Miron, Camelia-Elena Iurciuc Tincu, Lutfun Nahar, Satyajit D. Sarker, Anca Miron

**Affiliations:** 1 Faculty of Pharmacy, Grigore T. Popa University of Medicine and Pharmacy, 16, Universitatii Street, 700115 Iasi, Romania; lungu.cristina@umfiasi.ro (C.L.); gafton.mihaela@d.umfiasi.ro (M.G.); camelia_tincu83@yahoo.com (C.-E.I.T.); 2 Institute of Biological Research Iasi, Branch of NIRDBS—National Institute of Research and Development for Biological Sciences, 47, Lascar Catargi Street, 700107 Iasi, Romania; cosmin-teodor.mihai@laboratorpraxis.ro (C.-T.M.); gabrielacapraru@yahoo.com (G.V.); daniela_gherghel@yahoo.com (D.G.); 3 Medical Investigations Praxis SRL, 35, Moara de Vant Street, 700376 Iasi, Romania; 4 Faculty of Chemistry, Alexandru Ioan Cuza University of Iasi, 11, Carol I Boulevard, 700506 Iasi, Romania; ionelm@uaic.ro; 5 Institute of Interdisciplinary Research—CERNESIM Centre, Alexandru Ioan Cuza University of Iasi, 11, Carol I Boulevard, 700506 Iasi, Romania; 6 Faculty of Medicine, Grigore T. Popa University of Medicine and Pharmacy, 16, Universitatii Street, 700115 Iasi, Romania; sorin.miron@umfiasi.ro; 7 Faculty of Chemical Engineering and Environmental Protection, Gheorghe Asachi Technical University of Iasi, 73, Prof. Dr. Docent Dimitrie Mangeron Street, 700050 Iasi, Romania; 8 Laboratory of Growth Regulators, Palacký University and Institute of Experimental Botany, The Czech Academy of Sciences, Šlechtitelů 27, 78371 Olomouc, Czech Republic; profnahar@outlook.com; 9 Centre for Natural Products Discovery, School of Pharmacy and Biomolecular Sciences, Liverpool John Moores University, Byrom Street, Liverpool L3 3AF, UK; s.sarker@ljmu.ac.uk

**Keywords:** *Pinus cembra* L., bark extract, resveratroloside, pinostilbenoside, HeLa cells, antioxidant activity, antitumor activity

## Abstract

Stilbenes are plant secondary metabolites with remarkable antidiabetic, anti-inflammatory, antimicrobial, antioxidant, antitumor, and neuroprotective properties. As these compounds are valuable constituents in healthcare products and promising drug candidates, exploring new sources of stilbenes is essential for therapeutic advancement. The present study reports the isolation of two stilbene glycosides, resveratroloside and pinostilbenoside, from *Pinus cembra* L. bark. Their antioxidant activity and cytotoxic effects against HeLa cells were evaluated in comparison to the raw bark extract. The structures of resveratroloside and pinostilbenoside were confirmed by nuclear magnetic resonance (NMR) and mass spectrometry (MS) data analyses. Antioxidant activity was assessed by 2,2-diphenyl-1-picrylhydrazyl (DPPH) radical scavenging and reducing power assays. Cell viability, apoptosis, cell proliferation, and cell cycle assays were used to evaluate the cytotoxic potential against HeLa cells. Resveratroloside and pinostilbenoside exhibited lower activity as free radical scavengers and reducing agents. However, they showed greater efficacy in reducing viability and suppressing proliferation in human cervical carcinoma HeLa cells. Given the promising findings of our study, the therapeutic potential of resveratroloside and pinostilbenoside should be further investigated.

## 1. Introduction

Stilbenes are secondary metabolites with a 1,2-diphenylethylene (C6-C2-C6) structure biosynthesized in plants as a response to various stress conditions, such as bacterial, fungal, and viral infections, insect attacks, UV radiation, and heat. Most of them act as phytoalexins, playing a key role in plant defense against various phytopathogens [1,2,3]. According to Teka et al. [2], 459 stilbenes from 45 plant families and 196 plant species have been identified to date. The plant families Cyperaceae, Dipterocarpaceae, Euphorbiaceae, Fabaceae, Gnetaceae, Moraceae, Orchidaceae, Pinaceae, Polygonaceae, and Vitaceae are recognized for their high stilbene content [2]. Stilbenes exhibit significant structural diversity arising from hydroxylation, methoxylation, prenylation, glycosylation, isomerization, and oligomerization. Due to the ethylene moiety, stilbenes exist in two stereoisomeric forms: *trans* (*E*)-stilbene and *cis* (*Z*)-stilbene, the former being more stable and more common in nature [1,2].

The vast structural diversity endows stilbenes with remarkable bioactivity and versatility [1]. Resveratrol (3,5,4′-trihydroxy-stilbene), the most prominent stilbene, acts on multiple pathways involved in oxidative stress, inflammation, and cell death, such as nuclear erythroid 2-related factor 2 (Nrf2), nuclear factor kappa-light-chain-enhancer of activated B cells (NF-κB), forkhead box O (FOXO), signal transducer and activator of transcription (STAT) 1/3, phosphatidylinositol 3-kinase/protein kinase B (PI3K/Akt), c-Jun NH 2-terminal kinase (JNK), adenosine monophosphate-activated protein kinase (AMPK), insulin-like growth factor 1 receptor (IGF-1R)/Akt/Wnt, and p53 pathways [2,4,5,6]. Additionally, resveratrol targets various enzymes, cytokines, chemokines, and adhesion molecules, exerting a positive impact on superoxide dismutase (SOD), glutathione peroxidase (GPx), glutathione *S*-transferase (GST), glutathione reductase (GR), and sirtuin 1/3 (SIRT1/3) while suppressing matrix metalloproteinase (MMP)-2, -9, myeloperoxidase (MPO), nicotinamide adenine dinucleotide phosphate oxidase 4 (NOX4), cyclooxigenases (COX), inducible nitric oxide synthase (iNOS), interleukin (IL)-1, -6, -8, tumor necrosis factor (TNF)-α, monocyte chemoattractant protein-1 (MCP-1), C-X-C motif chemokine ligand 10 (CXCL10), chemokine (C-C motif) ligand 3 (CCL3), intercellular adhesion molecule 1 (ICAM-1), and vascular cell adhesion molecules (VCAMs) [6,7]. In vitro and in vivo studies have revealed the wide range of biological activities of resveratrol (anti-aging, antidiabetic, anti-inflammatory, anti-obesity, anti-osteoporosis, antioxidant, antitumor, cardioprotective, and neuroprotective) [8]. Human clinical trials conducted in recent years have provided evidence for the benefits of resveratrol in diabetes as well as neurological and cardiovascular diseases [7]. Pterostilbene (3,5-dimethoxy-4′-hydroxystilbene), a dimethoxy analog of resveratrol, is another promising candidate for clinical use due to its anti-inflammatory, antioxidant, and antitumor effects. Some of its targets (AMPK, PI3K/Akt, Nrf2, STAT3, SIRT1, NF-κB, TNF- α, IL-1β, -6, MMP-2, -9, COX-2, SOD) are similar to those of resveratrol. Pterostilbene inhibits transforming growth factor (TGF)-1β (involved in fibrotic diseases). In tumor cells, pterostilbene induces autophagy and modulates metastasis-associated protein 1 (MTA1)/hypoxia-inducible factor 1 α (HIF1α) and phosphatase and tensin homolog (PTEN)/Akt pathways (involved in cell proliferation, angiogenesis, and cell growth, respectively), microRNAs (miRNAs), endoplasmic reticulum stress (a limiting factor in tumor development), and epithelial–mesenchymal transition (involved in cell invasion) [9]. Piceatannol (3,3′,4,5′-tetrahydroxy-stilbene), a hydroxylated analog of resveratrol, is another stilbene with pleiotropic effects that interacts with various pathways (Janus kinase (JAK)/STAT, Nrf2, NF-κB, FOXO, PI3K/Akt) and molecular/cellular targets (mammalian target of rapamycin (mTOR), epidermal growth factor receptor (EGFR), activator protein-1 (AP-1), p38-mitogen-activated protein kinase (MAPK), SIRT1, STAT3, TGF-β) [10]. Pinosylvin (3,5-dihydroxy-stilbene) possesses a broad spectrum of biological activities, e.g., anti-inflammatory, antimicrobial, antioxidant, antitumor, and neuroprotective, due to its ability to interact with several targets associated with various diseases [Nrf2/antioxidant response element (ARE), PI3K/Akt-glycogen synthase kinase-3β (GSK-3β), focal adhesion kinase (FAK)/cellular Src (c-Src)/extracellular signal-regulated kinase (ERK), and p38 signaling pathways, COX-2, MMP-2, -9, iNOS, IL-6] [11]. Another stilbene of considerable interest is isorhapontigenin (3,5,4′-trihydroxy-3′-methoxystilbene), which is notable for its anti-inflammatory, antioxidant, and antitumor activities mainly attributed to the modulation of EGFR-PI3K-Akt, NF-κB, and Nrf2 pathways [12].

Many other stilbenes, both monomers and oligomers, have been investigated for their biological activities and the mechanisms supporting their bioactivities. According to Teka et al. [2], the bioactivity of 116 stilbenes has been investigated to date; the antidiabetic, anti-inflammatory, antimicrobial, antioxidant, antitumor, and neuroprotective effects are the frequently reported activities [2]. Overall, the stilbene scaffold has shown an outstanding biological potential. Nowadays, stilbenes are valuable ingredients in dietary supplements, functional foods, and cosmetic products [1,2,13,14] and some of them (resveratrol, pterostilbene) are undergoing clinical trials to evaluate their benefits in severe diseases [1]. Therefore, the exploration of novel stilbene sources is crucial for therapeutic progress.

Some of the above-mentioned stilbenes have also been identified in the bark of conifer species, for example, resveratrol in the bark of *Picea abies* (L.) Karst. [15,16], *Picea mariana* (Mill.) Britton, Sterns & Poggenb. [17], and *Pinus koraiensis* Siebold & Zucc. [18], piceatannol in the bark of *Picea abies* (L.) Karst. [15,16], pinosylvin and its monomethyl and dimethyl ethers in the bark of *Picea glauca* (Moench) Voss., *Pinus resinosa* Sol. ex Aiton, and *Pinus banksiana* Lamb. [19], and isorhapontigenin in the bark of *Picea abies* (L.) Karst. [15] and *Picea mariana* (Mill.) Britton, Sterns & Poggenb. [17]. To the best of our knowledge, the presence of stilbenes in the bark of *Pinus cembra* L. has not been investigated before. Stilbene derivatives (pinostilbene, pinosylvin and its monomethyl and dimethyl ethers, dihydropinosylvin and its monomethylether) have been reported only in the knotwood and heartwood of this species [20,21]. In the present study, we report for the first time the presence of two stilbene glycosides, resveratroloside and pinostilbenoside, in *Pinus cembra* L. bark as well as their antioxidant activity and cytotoxicity on human cervical carcinoma HeLa cells.

## 2. Results

### 2.1. Structure Elucidation of Compounds ***1*** and ***2***

**Figure 1:**
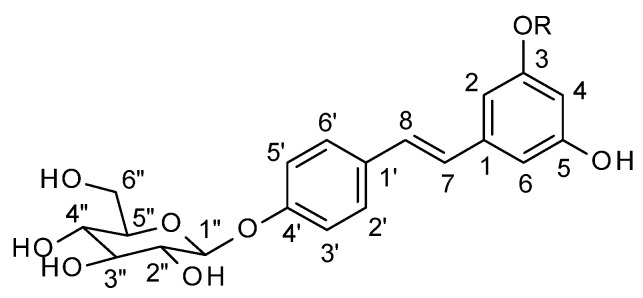
Stilbene glycosides isolated from *Pinus cembra* L. bark [resveratroloside (**1**) R = H, pinostilbenoside (**2**) R = Me].

The isolated compounds were identified as *trans*-resveratroloside (*trans*-resveratrol 4′-*O*-β-D-glucopyranoside, compound **1**) and *trans*-pinostilbenoside (*trans*-pinostilbene 4′-*O*-β-D-glucopyranoside, compound **2**) (Figure 1) by nuclear magnetic resonance (NMR) spectroscopy, notably, ^1^H NMR and ^13^C NMR, and by comparison of their ^1^H-NMR and ^13^C-NMR data with the literature data [18,22,23,24,25]. The molecular weights of compounds **1** and **2** were determined using high-resolution electrospray ionization mass spectrometry in positive ion mode (HRESIMS).

*Trans*-resveratroloside (*trans*-3,5,4′-trihydroxystilbene 4′-*O*-β-D-glucopyranoside, compound **1**): amorphous, white powder; HRESIMS *m*/*z* 391.1383 [M + H]^+^ (calculated 391.1387 for C_20_H_23_O_8_); ^1^H NMR (400 MHz, CD_3_OD) δ 7.45 (2H, d, *J* = 8.8 Hz, H-2′,6′), 7.08 (2H, d, *J* = 8.8 Hz, H-3′,5′), 7.00 (1H, d, *J* = 16.4 Hz, H-8), 6.88 (1H, d, *J* = 16.4 Hz, H-7), 6.47 (2H, d, *J* = 2.0 Hz, H-2,6), 6.18 (1H, dd, *J* = 2.0 Hz, H-4), glucose 4.91 (1H, d, *J* = 7.6 Hz, H-1″), 3.91 (1H, dd, *J* = 12.0,1.6 Hz, H-6″a), 3.71 (1H, dd, *J* = 12.0, 5.2 Hz, H-6″b), 3.38–3.50 (4H, overlapped peaks as multiplet, H-2″,3″,4″,5″); ^13^C NMR (100 MHz, CD_3_OD) δ 159.9 (C-3,5), 158.8 (C-4′), 141.2 (C-1), 133.4 (C-1′), 129.0 (C-8), 128.73 (C-2′,6′), 128.7 (C-7), 118.1 (C-3′,5′), 106.1 (C-2,6), 103.1 (C-4), glucose 102.4 (C-1″), 78.3 (C-3″), 78.2 (C-5″), 75.1 (C-2″), 71.5 (C-4″), 62.7 (C-6″).

*Trans*-pinostilbenoside (*trans*-3-methoxy-5,4′-dihydroxystilbene 4′-*O*-β-D-glucopyranoside, compound **2**): amorphous, white powder; HRESIMS *m*/*z* 405.1538 [M + H]^+^ (calculated 405.1544 for C_21_H_25_O_8_; ^1^H NMR (400 MHz, CD_3_OD) δ 7.47 (2H, d, *J* = 8.8 Hz, H-2′,6′), 7.09 (2H, d, *J* = 8.8 Hz, H-3′,5′), 7.04 (1H, d, *J* = 16.4 Hz, H-8), 6.93 (1H, d, *J* = 16.4 Hz, H-7), 6.58 (1H, d, *J* = 1.6 Hz, H-2), 6.57 (1H, d, *J* = 1.6 Hz, H-6), 6.27 (1H, dd, *J* = 2.0 Hz, H-4), glucose 4.93 (1H, d, *J* = 7.2 Hz, H-1″), 3.91 (1H, dd, *J* = 12.0,1.6 Hz, H-6″a), 3.78 (3H, s, 3-OMe), 3.71 (1H, dd, *J* = 12.0, 5.2 Hz, H-6″b), 3.39–3.48 (4H, overlapped peaks as multiplet, H-2″,3″,4″,5″); ^13^C NMR (100 MHz, CD_3_OD) δ 162.7 (C-3), 159.9 (C-5), 158.9 (C-4′), 141.2 (C-1), 133.3 (C-1′), 129.3 (C-8), 128.8 (C-2′,6′), 128.6 (C-7), 118.1 (C-3′,5′), 107.0 (C-6), 104.7 (C-2), 101.8 (C-4), glucose 102.4 (C-1″), 78.3 (C-3″), 78.2 (C-5″), 75.1 (C-2″), 71.5 (C-4″), 62.7 (C-6″), 55.8 (3-OMe).

The NMR and HRESIMS spectra are provided in the Supplementary Materials.

### 2.2. Antioxidant Activity

Resveratroloside (**1**) and pinostilbenoside (**2**) were less active than the raw bark extract and positive control, catechin, in 2,2-diphenyl-1-picrylhydrazyl (DPPH) radical scavenging and reducing power assays. The antioxidant effects of the raw bark extract and catechin have been reported elsewhere [26]. At 166.67 μg/mL, resveratroloside (**1**) and pinostilbenoside (**2**) scavenged DPPH radical by 19.88 ± 0.97% and 14.67 ± 0.51%, respectively, while the scavenging effects of the raw bark extract and catechin were significantly higher (72.32 ± 0.69% and 95.74 ± 0.05%, respectively). Similarly, at 50 μg/mL, resveratroloside (**1**) and pinostilbenoside (**2**) had weaker reducing effects (0.49 ± 0.00 and 0.09 ± 0.01, respectively) in comparison with the raw bark extract and catechin (0.90 ± 0.00 and 0.53 ± 0.00, respectively). It is noteworthy that, at 50 μg/mL, the reducing capacity of resveratroloside (**1**) was slightly lower than that of catechin.

### 2.3. Cytototoxic Activity on HeLa Cells

#### 2.3.1. Effects on Cell Viability

**Figure 2:**
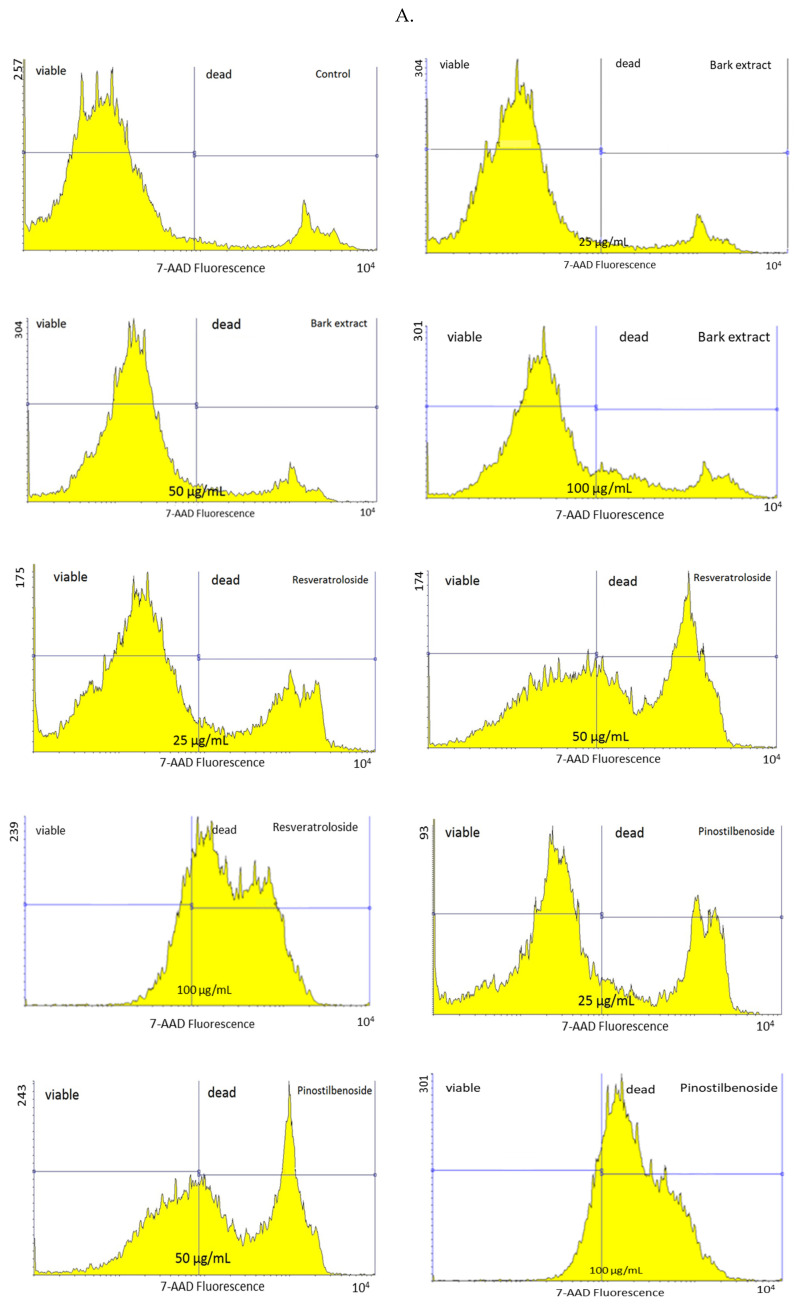

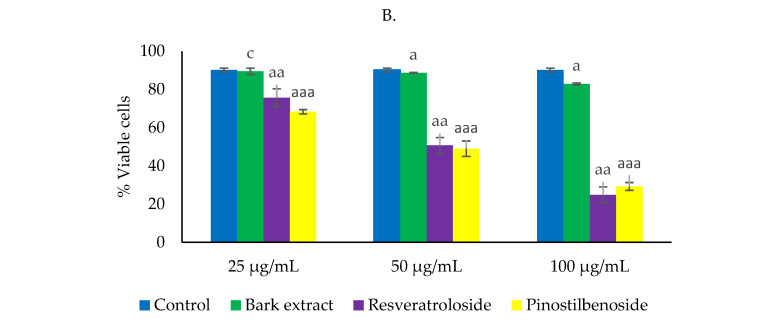
Viability of HeLa cells after 48 h exposure to the raw bark extract, resveratroloside (**1**), and pinostilbenoside (**2**), assessed by 7-amino-actinomycin (7-AAD) staining ((**A**)—histograms; (**B**)—HeLa cell viability); (a) *p* < 0.001, (c) *p* > 0.05.

After 48 h incubation, resveratroloside (**1**) and pinostilbenoside (**2**) strongly reduced HeLa cell viability as compared to the control and raw bark extract. At 25 μg/mL, resveratroloside (**1**) and pinostilbenoside (**2**) decreased the percentage of viable cells to 75.67 ± 4.68% and 68.36 ± 1.14%, respectively. HeLa cell viability dropped to near 50% in the case of exposure to 50 μg/mL of resveratroloside (**1**) or pinostilbenoside (**2**). At 100 μg/mL, both resveratroloside (**1**) and pinostilbenoside (**2**) caused significant reductions in HeLa cell viability (24.82 ± 4.20% and 29.73 ± 0.41%, respectively). Approximately 90% of the HeLa cells were viable in the control and cultures treated with the raw bark extract at 25 and 50 μg/mL. HeLa cell viability was slightly reduced to 82.91 ± 0.47% by the raw bark extract at 100 μg/mL (Figure 2).

#### 2.3.2. Effects on Apoptosis

**Figure 3:**
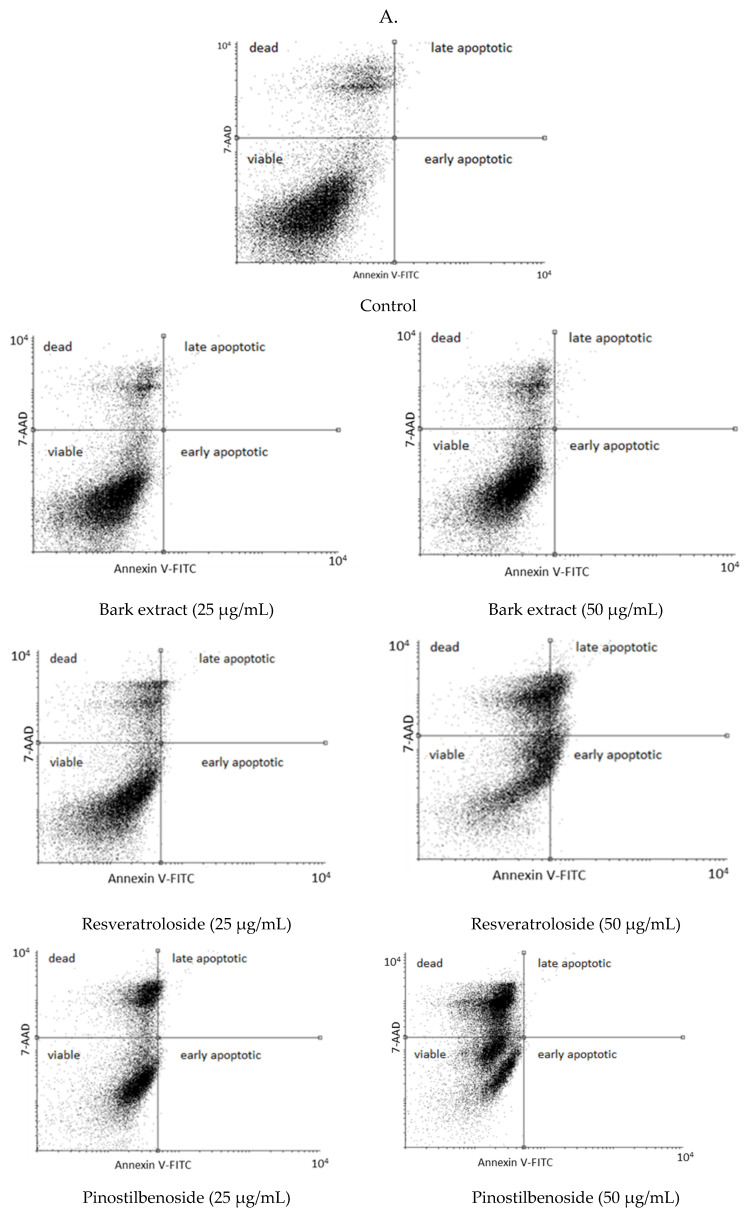

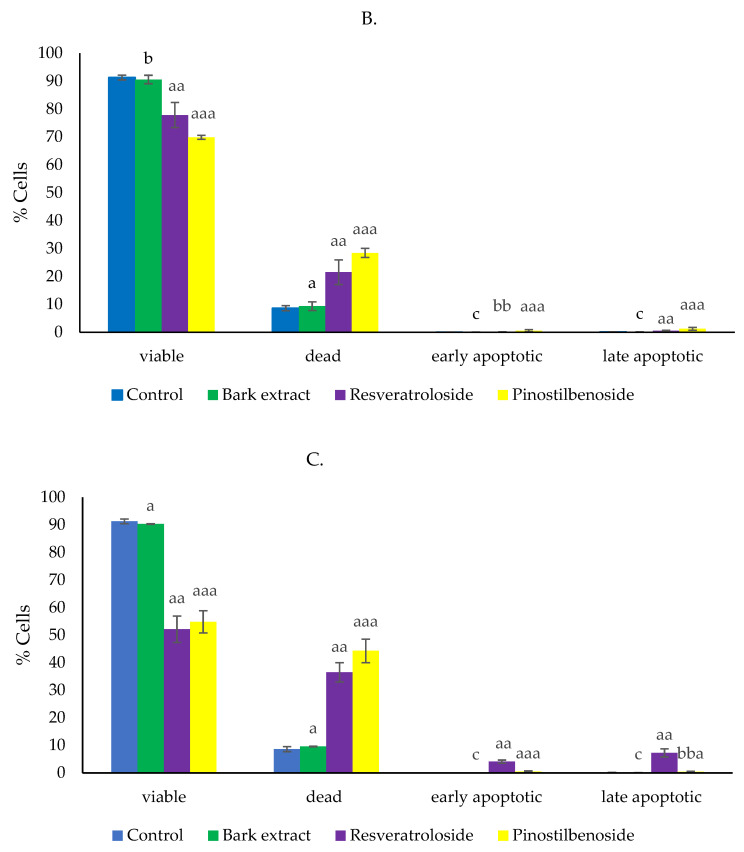
Percentage distribution of viable, dead, early apoptotic, and late apoptotic HeLa cells after 48 h exposure to the raw bark extract, resveratroloside (**1**), and pinostilbenoside (**2**) at 25 μg/mL (**A**,**B**) and 50 μg/mL (**A**,**C**), assessed by annexin V–fluorescein isothiocyanate (FITC)/7-amino-actinomycin (7-AAD) staining ((**A**)—cytograms; (**B**,**C**)—HeLa cell distribution); (a) *p* < 0.001, (b) *p* < 0.05; (c) *p* > 0.05.

Annexin V-fluorescein isothiocyanate (FITC)/7-amino-actinomycin (7-AAD) staining indicated no apoptosis-inducing effects for the raw bark extract. The percentages of early and late apoptotic cells after 48 h exposure to the raw bark extract (at 25 μg/mL: 0.01 ± 0.01% and 0.11 ± 0.01%, respectively; at 50 μg/mL: 0.02 ± 0.01% and 0.09 ± 0.02%, respectively) were similar to the control (0.01 ± 0.01% and 0.10 ± 0.03%, respectively). On the other hand, resveratroloside (**1**) triggered apoptosis in HeLa cells, the effect being more pronounced at 50 μg/mL (4.06 ± 0.58% early apoptotic cells and 7.25 ± 1.46% late apoptotic cells vs. 0.09 ± 0.03% early apoptotic cells and 0.54 ± 0.17% late apoptotic cells in HeLa cells exposed to resveratroloside (**1**) at 25 μg/mL). The pro-apoptotic effects of pinostilbenoside (**2**) were negligible (at 25 μg/mL: 0.54 ± 0.37% early apoptotic cells and 1.22 ± 0.52% late apoptotic cells; at 50 μg/mL: 0.45 ± 0.30% early apoptotic cells and 0.37 ± 0.17% late apoptotic cells). In addition, resveratroloside (**1**) and pinostilbenoside (**2**) dose-dependently augmented the percentage of dead cells (21.53 ± 4.42% and 36.52 ± 3.53% in HeLa cells exposed to 25 and 50 μg/mL of resveratroloside (**1**), respectively; 28.41 ± 1.63% and 44.32 ± 4.28% in HeLa cells exposed to 25 and 50 μg/mL of pinostilbenoside (**2**), respectively) (Figure 3).

#### 2.3.3. Effects on Cell Cycle

**Figure 4:**
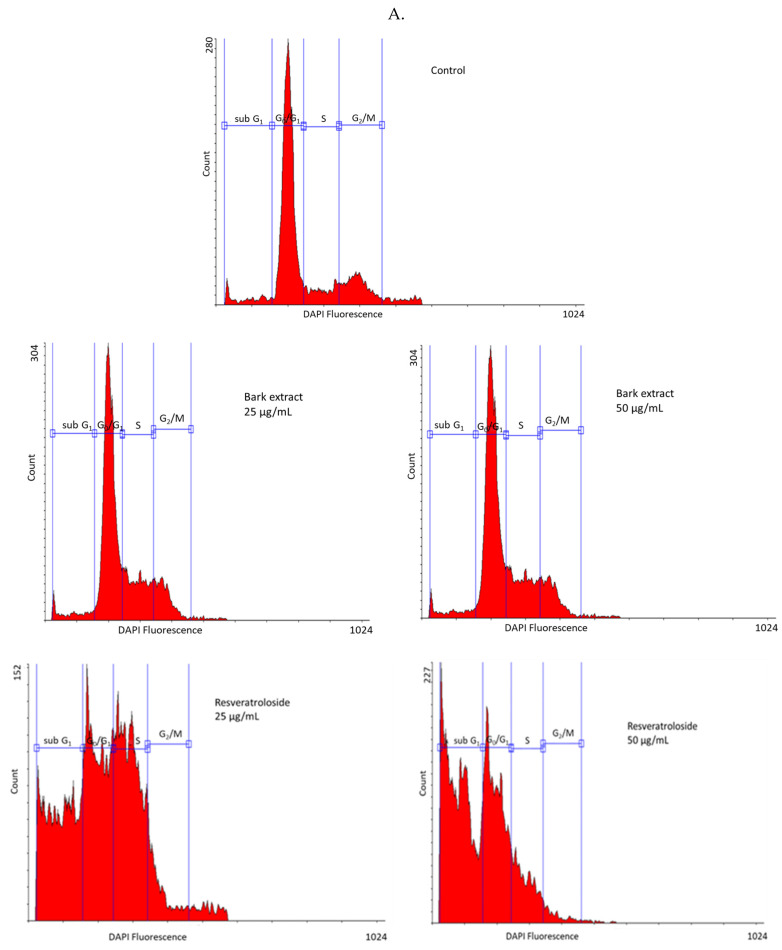

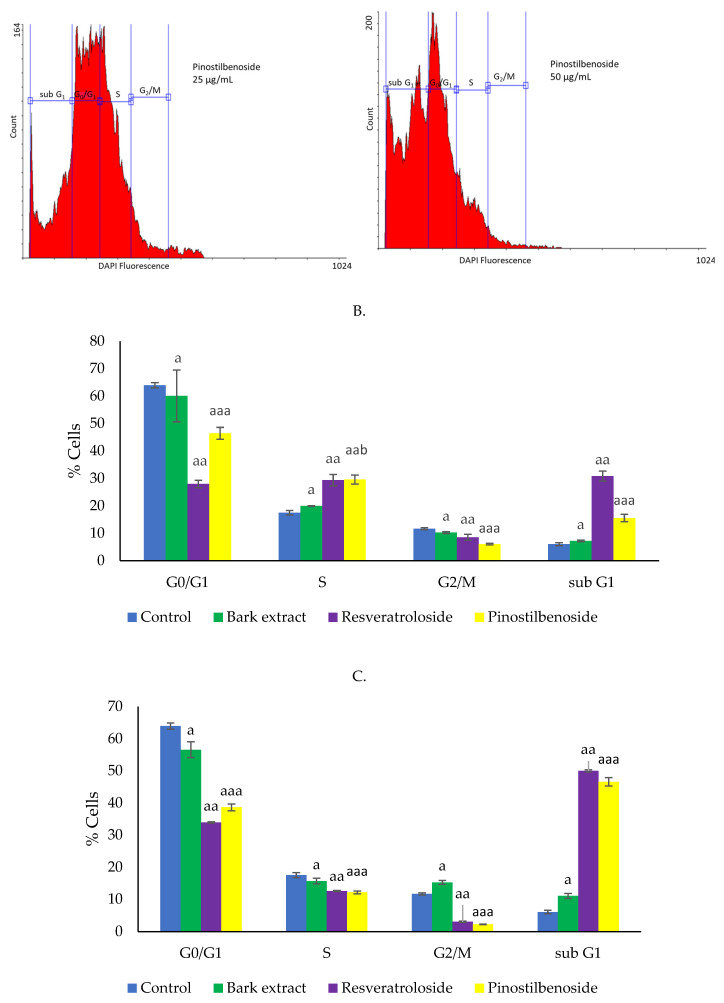
Cell cycle analysis in HeLa cells after 48 h exposure to the raw bark extract, resveratroloside (**1**), and pinostilbenoside (**2**) at 25 μg/mL (**A**,**B**) and 50 μg/mL (**A**,**C**), assessed by nuclear isolation medium—4′,6-diamidino-2-phenylindole dihydrochloride (NIM-DAPI) staining ((**A**)—histograms; (**B**,**C**)—cell cycle distribution); (a) *p* < 0.001; (b) *p* < 0.05.

As shown in Figure 4, in the control, HeLa cells were distributed in high percentage in the G0/G1 phase (63.93 ± 0.94%) and, to a lesser extent, in the S and G2/M phases (17.53 ± 0.78% and 11.67 ± 0.35%, respectively); a small percentage of cells (6.03 ± 0.55%) were in the sub-G1 phase. At 25 μg/mL, the raw bark extract induced a slight increase in the percentage of HeLa cells in the S phase (19.96 ± 0.15% vs. 17.53 ± 0.78% in the control) whereas, at 50 μg/mL, modest accumulations of cells in the G2/M and sub-G1 phases were observed (15.25 ± 0.62% vs. 11.67 ± 0.35% in the control and 11.03 ± 0.75% vs. 6.03 ± 0.55% in the control, respectively). At 25 μg/mL, resveratroloside (**1**) caused a marked increase in the percentages of HeLa cells in the S and sub-G1 phases (29.39 ± 2.07% vs. 17.53 ± 0.78% in the control and 30.88 ± 1.86% vs. 6.03 ± 0.55% in the control, respectively). A similar behavior was noticed for pinostilbenoside (**2**). Following 48 h exposure to 25 μg/mL pinostilbenoside (**2**), the percentages of HeLa cells in the S and sub-G1 phases increased in comparison to the control (29.61 ± 1.62% and 15.56 ± 1.35%, respectively, vs. 17.53 ± 0.78% and 6.03 ± 0.55%, respectively). A strong accumulation in the sub-G1 phase was observed in HeLa cells after 48 h treatment with 50 μg/mL resveratroloside (**1**) and pinostilbenoside (**2**) (49.96 ± 0.36% and 46.56 ± 1.31%, respectively, vs. 6.03 ± 0.55% in the control).

#### 2.3.4. Effects on Cell Proliferation

**Figure 5:**
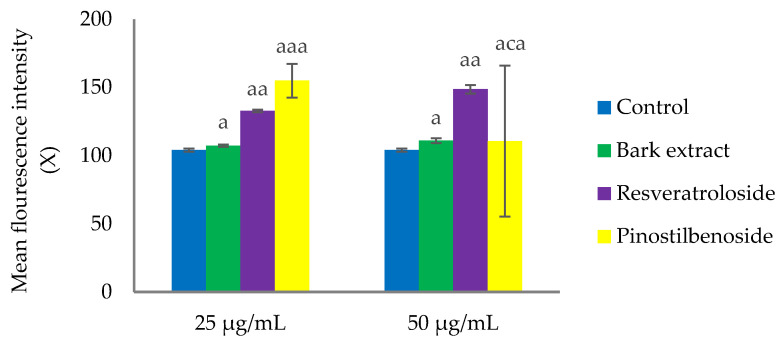
Mean fluorescence intensity in HeLa cells after 48 h exposure to the raw bark extract, resveratroloside (**1**), and pinostilbenoside (**2**), assessed by carboxyfluorescein succinimidyl ester (CFSE) staining; (a) *p* < 0.001, (c) *p* > 0.05.

The raw bark extract (25 and 50 µg/mL) had negligible effects on HeLa cell proliferation as indicated by minor increases in the mean fluorescence intensity (X) of the 48 h-treated cells compared to the control cells (107.30 ± 0.79 and 110.91 ± 1.78, respectively, vs. 104.06 ± 1.11). In contrast to the raw extract, exposure to both concentrations of resveratroloside (**1**) caused significant reductions in HeLa cell proliferation (X values of 132.81 ± 0.96 and 148.65 ± 3.13, respectively, vs. 104.06 ± 1.11 in the control). In the case of pinostilbenoside (**2**), only the low dose (25 µg/mL) exerted an antiproliferative effect (X value of 154.96 ± 12.35 vs. 104.06 ± 1.11 in the control) (Figure 5).

## 3. Discussion

Conifer bark, the medicinal use of which dates back more than 2000 years, contains bioactive alkaloids, flavonoids, lignans, phenolic acids, proanthocyanidins, stilbenes, and terpenoids that contribute to its therapeutic potential. Various conifer bark extracts are used nowadays in the nutraceutical, food, and pharmaceutical industries because of their health-promoting effects in numerous ailments and diseases [27,28,29]. Such extracts are Pycnogenol^®^, Flavangenol^®^, and Oligopin^®^ derived from *Pinus pinaster* Ait. bark [29], Enzogenol^®^ produced from *Pinus radiata* D. Don bark [30], and Abigenol^®^ originating from *Abies alba* Mill. bark [31].

*Pinus cembra* L. (Pinaceae, Swiss stone pine, Arolla pine, cembran pine, cedar pine) is a coniferous tree growing in the Alps and Carpathian Mountains [32]. The bark has been scarcely investigated for its chemical composition and biological activity. We have previously assessed the antioxidant potential of the raw bark extract (80% methanolic bark extract) and found EC_50_ values of 71.1 ± 0.5 and 26.0 ± 0.3 μg/mL in the DPPH radical scavenging and reducing power assays, respectively. The antioxidant potential of the raw bark extract is strongly associated with the total phenolic content, quantified as 299.3 ± 1.4 mg/g. In the same assays, catechin was more effective (EC_50_ = 5.56 ± 0.05 and 3.70 ± 0.03 μg/mL, respectively) [26]. Other polar conifer bark extracts (80% methanolic, aqueous) scavenged the DPPH radical with EC_50_ values ranging from 6.46 ± 0.36 to 100.1 ± 0.1 μg/mL [29,33,34,35,36]. In the reducing power assay, polar conifer bark extracts exhibited EC_50_ values ranging from 9.17 ± 0.13 to 25.32 ± 0.62 μg/mL [29,35,37]. Overall, the EC_50_ values of the raw extract of cembran pine bark in the DPPH and reducing power assays fall within the range of values reported for other polar conifer bark extracts.

The cytotoxic potential of the raw bark extract was further investigated. HeLa cells were used for this purpose as they have advantages over other tumor cell lines, for example, high adaptive capacity and proliferation rate [38]. The study revealed moderate or weak cytotoxicity. At 25 and 50 μg/mL, the extract had an insignificant impact on the viability of HeLa cells, lacked apoptosis-inducing effects, and induced slight increases (≤5%) in the HeLa cell percentages in the S, G2/M, and sub-G1 phases in comparison with the control. Other polar conifer bark extracts exhibited higher activity on HeLa cells. The aqueous extract of *Pinus massoniana* Lamb. bark significantly inhibited HeLa cell viability and caused a substantial increase in the proportions of HeLa cells in the sub-G1 and G2/M phases [39]. Accumulation of HeLa cells in the sub-G1 phase is considered an indicator of a pro-apoptotic effect [39,40]. In other studies, the extract significantly inhibited the migration and invasion of HeLa cells, respectively, the latter effect being attributed to cathepsin B down-regulation [41,42]. Pro-apoptotic effects in HeLa cells were also reported for the 80% methanolic extract of *Pinus sylvestris* L. bark [43], ethanolic extract of *Pinus merkusii* Jung. & de Vriese bark [44], and procyanidin-rich extract of *Pinus koraiensis* Siebold & Zucc. bark [45]. The pro-apoptotic effects of conifer bark extracts were found to be mediated by activation of caspase-9 and -3, up-regulation of the pro-apoptotic protein Bax, and down-regulation of the anti-apoptotic protein Bcl-2 and survivin [39,44,45].

Purification of the cembran pine raw bark extract resulted in the isolation of two stilbene glycosides, namely resveratroloside (**1**) and pinostilbenoside (**2**), the structures of which were confirmed through spectroscopic techniques. To the best of our knowledge, this is the first report on the presence of resveratroloside (**1**) and pinostilbenoside (**2**) in *Pinus cembra* L. bark. Both compounds were previously isolated from other conifer barks: resveratroloside from *Pinus sibirica* R. Mayr bark [46] and pinostilbenoside from *Pinus sibirica* R. Mayr bark [46] and *Pinus koraiensis* Siebold & Zucc. bark [18].

The evaluation of the antioxidant potential of resveratroloside (**1**) and pinostilbenoside (**2**) demonstrated weaker effects than the raw bark extract, indicating a potential synergistic interaction among the components of the extract. Previous studies have reported similar synergistic interactions in pine bark extracts. Pycnogenol, a standardized extract obtained from the bark of French maritime pine (*Pinus maritima* Lam.), exhibits stronger biological effects than its components when tested individually [27]. In contrast to our findings, Dar et al. (2016) reported a strong antioxidant potential for resveratroloside in the DPPH assay (IC_50_ = 14.0 μg/mL) [47]. The explanation lies in the fact that Dar et al. [47] used another experimental protocol. Resveratroloside (**1**) exhibited higher activity than pinostilbenoside (**2**) in both assays. The findings align with previous studies reporting a higher antioxidant capacity (evaluated as oxygen radical absorbance capacity, ORAC) for resveratroloside (4.01 ± 0.71 Trolox equivalents/μM) than pinostilbenoside (1.89 ± 0.25 Trolox equivalents/μM). In the same assay, the aglycones, resveratrol and pinostilbene, were more active than the corresponding glycosides, showing ORAC values of 5.26 ± 0.26 and 5.01 ± 0.27 Trolox equivalents/μM, respectively [48]. Glycosylation and methylation negatively impact the antioxidant capacity of stilbenes by blocking the free phenolic hydroxyl groups responsible for the antioxidant activity [49]. On the other hand, glycosylation and methylation of the stilbene hydroxyl groups might enhance other bioactivities such as tyrosinase inhibitory activity [48] and anticancer activity, respectively [3]. Glycosylation enhances the stability of stilbenes, while methylation increases their lipophilicity, leading to improved bioavailability [3].

In this study, resveratroloside (**1**) and pinostilbenoside (**2**) demonstrated promising cytotoxic activity against HeLa cells. The activity was evaluated after 48 h of incubation with 25 or 50 μg/mL of each compound (equivalent to 64 or 128 μM of resveratroloside (**1**) and 62 or 124 μM of pinostilbenoside (**2**)). The selection of the concentrations to be tested and incubation time was based on previous studies investigating the cytotoxicity of resveratrol in HeLa cells [50,51,52]. In addition, this study revealed pronounced cytotoxicity (less than 30% cell viability) for both compounds at 100 μg/mL. This served as additional support for selecting lower doses (25 and 50 μg/mL) in cell-based assays. To the best of current knowledge, this is the first study evaluating the effects of resveratroloside (**1**) and pinostilbenoside (**2**) on human cervical carcinoma HeLa cells. Resveratroloside (**1**) has been scarcely investigated for its antitumor potential. Only its antiproliferative effects on H2452 malignant pleural mesothelioma cells (approximately 30% inhibition at 200 μM) were reported so far [53]. To the best of our knowledge, the antitumor potential of pinostilbenoside (**2**) has not been investigated before.

Cytotoxic therapies eliminate cancer cells by triggering various pathways of cell death. Induction of apoptosis (programmed cell death) has been a primary objective in cancer therapy for more than 30 years [54]. In recent years, many drugs, including natural compounds, have been reported to trigger other types of death in cancer cells such as autophagy, ferroptosis, necroptosis, pyroptosis, paraptosis, lysosome-dependent cell death, oncosis, and necrosis [55,56]. Resveratroloside (**1**) and pinostilbenoside (**2**) are not the sole stilbenes that cause tumor cell death by triggering non-apoptotic mechanisms. Resveratrol was reported to induce tumor cell death by apoptosis, autophagy, necroptosis, and necrosis [56,57]. Pterostilbene was found to activate apoptosis, autophagy, and necrosis in cancer cells, apoptosis being the major mechanism involved in cancer cell death [58]. Combrestatins (diaryl stilbenoids) are effective promoters of tumor necrosis [59].

Dysregulation of the cell cycle, a process involving cell growth, DNA replication, and cell division, is a hallmark of cancer. An important strategy in cancer therapy is the induction of cell cycle arrest. Flavopiridol, abemaciclib, and palbociclib are a few examples of antitumor drugs that suppress the cell cycle via inhibition of enzymes/proteins (cyclin-dependent kinases/cyclins) responsible for driving the progression of the cell cycle from one phase to the next one [60]. According to this study, resveratroloside (**1**) and pinostilbenoside (**2**) impeded HeLa cell proliferation, with pinostilbenoside (**2**) being more active than resveratroloside (**1**) at 25 μg/mL. This result aligns with previous studies showing that stilbenes inhibit the proliferation of tumor cell lines. Resveratrol [57], piceatannol [10], pterostilbene [61,62,63], and polydatin (piceid) [64] were reported to suppress the proliferation of various cancer cell lines (lung, prostate, breast, colorectal, liver, pancreatic, cervical, ovarian, bladder, leukemia, multiple myeloma, bone, oral, esophageal, head and neck).

Regarding the impact of resveratroloside (**1**) and pinostilbenoside (**2**) on the HeLa cell cycle, both compounds induced a significant dose-dependent increase in the sub-G1 phase population. In addition, both compounds (25 μg/mL) induced cell cycle arrest at the S phase, indicating a blockage of DNA replication [60]. As mentioned earlier, the sub-G1 population is a hallmark of apoptosis [39,40]. In fact, not only apoptotic cells accumulate in the sub-G1 phase, but this phase consists of cells showing DNA fragmentation, a process observed in both apoptosis and necrosis [65,66,67]. When exploring a potential pro-apoptotic effect, only resveratroloside (**1**) showed activity (approximately 11% increase in the early and late apoptotic HeLa cells following 48 h treatment with resveratroloside (**1**) at 50 μg/mL). The results of this study indicate that resveratroloside (**1**) and pinostilbenoside (**2**) impact the viability and proliferation of HeLa cells by triggering mainly non-apoptotic (highly likely necrotic) cell death, as well as S-phase cell cycle arrest. Similar results have been reported for other stilbene derivatives. Resveratrol was found to induce apoptosis and block cell cycle progression in the S phase in human SW480 colon carcinoma, MCF7 breast carcinoma, HCE7 esophageal squamous carcinoma, HL60 promyelocytic leukemia cells [68], and neuro-2a cells derived from C1300 murine neuroblastoma [69]. Piceatannol caused apoptosis and G0/G1 phase arrest in T24 and HT1376 human bladder cancer cells [70]. Pterostilbene was reported to induce apoptosis and S-phase arrest in MOLT4 human leukemia cells [71], Jurkat and Hut-78 T-cell leukemia/lymphoma cells [72], and diffuse large B-cell lymphoma cells [73], apoptosis and G1 phase arrest in HT-29 colon cancer cells [58] and human gastric carcinoma AGS cells [74], and autophagy and S phase arrest in HCCC-9810 and RBE human cholangiocarcinoma cells [62].

To conclude the cytotoxicity assays, resveratroloside (**1**) and pinostilbenoside (**2**) reduced viability (mostly via non-apoptotic routes) and proliferation (via sub-G1- and S-phase arrest) in HeLa cells. The results are consistent with previous studies on the antitumor potential of stilbenes. Resveratrol, the basic scaffold of resveratroloside (**1**) and pinostilbenoside (**2**), was reported to promote cell cycle arrest at the S phase, apoptosis, and autophagy in HeLa cells [52]. Polydatin, a glycoside of resveratrol, namely resveratrol-3-*O*-β-mono-D-glucoside, reduced proliferation and induced apoptosis in HeLa cells [64]. In this study, resveratroloside (**1**) and pinostilbenoside (**2**) exhibited comparable activity in arresting the HeLa cell cycle at the S and sub-G1 phases (at 25 and 50 μg/mL, respectively). On the other hand, pinostilbenoside (**2**) exhibited higher activity than resveratroloside (**1**) in increasing the number of dead cells through non-apoptotic mechanisms (at 25 and 50 μg/mL) and in reducing HeLa cell proliferation (at 25 μg/mL). The latter findings are consistent with earlier studies reporting increased cytotoxic activity for the methoxylated analogs of resveratrol compared to resveratrol itself [75].

The two compounds (**1** and **2**) isolated in this study are stilbene glycosides. Glycosylation is known to positively impact the water solubility, intestinal absorption, and bioactivity of stilbenes [76,77]. A notable example is polydatin, one of the main compounds in the roots of *Polygonum cuspidatum* Sieb. et Zucc., identified in other plant species across the Liliaceae, Fabaceae, and Vitaceae families. Based on its anti-inflammatory, antioxidant, and apoptosis-modulating potential, polydatin displays diverse biological activities (anticancer, antidiabetic, antimicrobial, cardioprotective, hepatoprotective, and neuroprotective effects, as well as protective effects on the gastrointestinal, renal, respiratory, and skeletal systems). A large number of studies conducted on polydatin has revealed versatility in modulating numerous targets related to oxidative stress (Nrf2 and Akt pathways, glutathione, catalase (CAT), SOD, GPx, GST, MPO), inflammation (NF-κB, phospholipase A2 (PLA2), COX-2, iNOS, TNF-α, IL-1β, IL-6, ICAM-1, MAPKs, ERK1/2, JNK1/2), and apoptosis (p53/MAPK/JNK and PI3K/Akt/mTOR pathways, B-cell lymphoma 2 (Bcl-2), Bcl-2-associated x (Bax), D-cyclins, caspase-3, cytochrome c). Clinical trials support the benefits of polydatin in chronic pelvic pain, liver diseases, inflammatory bowel syndrome, and EGFR-tyrosine kinase inhibitor (TKI)-related ashes. Moreover, various drug delivery systems (liposomes, micelles, nanoparticles, polymeric nanocapsules) have been developed to improve the bioavailability, biocompatibility, and efficacy of polydatin [64,78].

The results of the present study, along with the remarkable biological potential of the stilbene scaffold and the broad bioactivity of polydatin, a resveratrol glycoside, indicate that the bioactive properties of resveratroloside (**1**) and pinostilbenoside (**2**) require further in-depth investigation. Future studies should explore the ability of resveratroloside (**1**) and pinostilbenoside (**2**) to modulate cellular signaling pathways, enzymes, and other molecules involved in the antioxidant defense and oxidative damage repair. Research on the antitumor potential (mechanisms underlying cytotoxic activity in HeLa cells, cytotoxicity against other tumor cell lines) should also continue. Exploration of additional bioactivities and development of appropriate delivery systems are crucial for the therapeutic valorization of resveratroloside (**1**) and pinostilbenoside (**2**).

## 4. Materials and Methods

### 4.1. Chemicals

Diethyl ether and ethyl acetate were purchased from Sigma-Aldrich Laborchemikalien GmbH (Seelze, Germany). Acetone, (+)-catechin, deuterated methanol (CD_3_OD), dimethylsulfoxide (DMSO), disodium hydrogen phosphate, DPPH radical, iron (III) chloride, methanol, polyamide 6 (50–160 µm), potassium ferricyanide, tetramethylsilane, and trichloroacetic acid were acquired from Sigma-Aldrich (Steinheim, Germany). Methanol for HPLC LiChrosolv^®^ and monosodium phosphate were from Merck KGaA (Darmstadt, Germany) while *n*-butanol was from Chimopar SA (Bucharest, Romania). Amphotericin B, Dulbecco’s Modified Essential Medium (DMEM), ethylenediaminetetraacetic acid (EDTA), fetal bovine serum, penicillin, phosphate-buffered saline (PBS), streptomycin, and trypsin were purchased from Biochrom AG (Berlin, Germany). The CellTrace^TM^ carboxyfluorescein succinimidyl ester (CFSE) cell proliferation kit was obtained from Invitrogen (Waltham, MA, USA). The annexin V-FITC/7-AAD apoptosis kit and nuclear isolation medium—4′,6-diamidino-2-phenylindole dihydrochloride (NIM-DAPI) were purchased from Beckman Coulter (Fullerton, CA, USA). Ultrapure water was obtained using the SG Water Ultra Clear TWF water purification system (Barsbüttel, Germany).

### 4.2. Plant Material

The source of plant material as well as drying and storage conditions have already been described elsewhere [26].

### 4.3. Isolation of Stilbene Glycosides

The dried bark fragments (150 g) were powdered and extracted with 80% aqueous methanol (1.5 L) by stirring with a magnetic stirrer for 1 h (500 rpm). The extraction was repeated twice. The combined extracts were filtered, evaporated under reduced pressure at 40 °C (Büchi R-210 rotary evaporator system, Büchi Labortechnik AG, Flawil, Switzerland), and freeze-dried (Unicryo TFD 5505 freeze-dryer, UniEquip GmbH, Munich, Germany), resulting in 23.09 g of raw bark extract (yield: 15.39%). The raw bark extract (21.16 g) was suspended in 210 mL of ultrapure water and extracted successively with diethyl ether (14 × 200 mL), ethyl acetate (10 × 200 mL), and *n*-butanol (8 × 200 mL). The resulting extracts were combined, evaporated under reduced pressure at 40 °C, and weighed to yield diethyl ether (8.16 g), ethyl acetate (3.45 g), and *n*-butanol (7.71 g) extractive fractions. The remaining aqueous phase was lyophilized yielding 1.80 g. The ethyl acetate extractive fraction (EAF, 1.5 g) was purified by open column chromatography (39 × 2.4 cm) using polyamide 6 as the stationary phase. Four separate fractions (EAF-1-4) were collected following elution with methanol–water 1:1 (*v*/*v*, 450 mL), methanol–water 7:3 (*v*/*v*, 300 mL), methanol (1100 mL), and acetone–water 7:3 (*v*/*v*, 1200 mL). Each fraction was evaporated under reduced pressure at 40 °C and lyophilized yielding 852.90, 135.30, 120.40, and 131.30 mg, respectively. Fraction EAF-1 (846.40 mg) was dissolved in methanol–water mixture (2:8, *v*/*v*, 14.5 mL) and further purified by semipreparative reversed-phase high-performance liquid chromatography (RP-HPLC) using an Agilent Technologies 1200 Series HPLC system (Agilent Technologies, Santa Clara, CA, USA) equipped with degasser (G1322A), quaternary pump (G1311A), thermostat (G1316A), and diode array detector (G1315B). The chromatographic separation was performed as follows: column: Discovery^®^ BIO Wide Pore C18 (250 × 100 mm, 5 μm), mobile phase: ultrapure water (A) and methanol (B), elution gradient: 0–5 min: 0% B, 5–15 min: 0–30% B, 15–45 min: 30–100% B, detection wavelength λ = 280 nm, flow rate 1 mL/min, and injection volume 1 mL. Twelve runs were performed and two fractions (EAF-1-1 and EAF-1-2) were collected corresponding to the retention times of 46–50 min and 59–61 min, respectively. The two fractions were evaporated under reduced pressure (40 °C) and lyophilized yielding 171 and 162.2 mg, respectively. EAF-1-1 (160 mg) was dissolved in methanol–water 1:1 (*v*/*v*, 12 mL) and purified by semipreparative RP-HPLC using the same conditions as previously mentioned except that the elution gradient was as follows: 0–35 min: 25% B, 35–45 min: 25–100% B, 45–55 min: 100% B. Compound **1** was collected in ten runs (retention time 13–31 min); the corresponding eluates were evaporated and freeze-dried yielding 58 mg. Another gradient (0–35 min: 40% B, 35–45 min: 40–100% B, 45–50 min: 100% B) applied to EAF-1-2 (160 mg dissolved in 9 mL of methanol–water 3.5:1.5, *v*/*v*) allowed the isolation of compound **2**. The eluates of eight runs, corresponding to the retention time of 16–30 min, were evaporated and lyophilized producing 84 mg of compound **2**.

### 4.4. Structure Elucidation

Structure elucidation of compounds **1** and **2** was performed by spectroscopic techniques including NMR spectroscopy and HRESIMS. ^1^H-NMR and ^13^C-NMR spectra were recorded on a Bruker Avance DRX 400 MHz NMR spectrometer (Bruker BioSpin, Rheinstetten, Germany). ^1^H- and ^13^C-NMR experiments were performed at 400 and 100 MHz, respectively. CD_3_OD was used to dissolve compounds **1** and **2**. Tetramethylsilane was used as an internal standard. The chemical shift values (δ) were expressed in ppm relative to tetramethylsilane. HRESIMS spectra were acquired in the positive ion mode on an LTQ Orbitrap with LTQ MS Mass Spectrometer (Thermo Fischer Scientific, Waltham, MA, USA).

### 4.5. Antioxidant Activity

#### 4.5.1. DPPH Radical Scavenging Assay

Resveratroloside (**1**) and pinostilbenoside (**2**) were dissolved in DMSO to achieve a concentration of 10 mg/mL and subjected to DPPH assay as previously described [26,79]. The assay is based on the ability of antioxidant agents to reduce the DPPH free radical (violet) to diphenylpicrylhydrazyl (DPPH-H, yellow) which causes a reduction in absorbance at 517 nm [79]. Briefly, each compound (10 mg/mL in DMSO, 0.05 mL) was mixed with a solution of DPPH radical in methanol (2.95 mL, A_517nm_ = 1.00 ± 0.05). The absorbance of the latter was determined before the compound was added (A_start_) and after a 5 min reaction time (A_end_). The DPPH radical scavenging activity (%) of each compound was calculated as 100 × (A_start_ − A_end_)/(A_start_) [26,79].

#### 4.5.2. Reducing Power Assay

Resveratroloside (**1**) and pinostilbenoside (**2**) were dissolved in DMSO (8.25 mg/mL). The reducing power assay was performed as previously reported [26,80,81]. The assay evaluates the ability of antioxidant agents to reduce potassium ferricyanide to potassium ferrocyanide, the latter being quantified as Perl’s Prussian Blue after reaction with ferric chloride (λ = 700 nm) [80,81]. In brief, each compound (8.25 mg/mL in DMSO, 0.1 mL) was mixed with 0.2 M phosphate buffer (pH = 6.6, 2.4 mL) and 1% potassium ferricyanide (2.5 mL) followed by 20 min incubation at 50 °C. The reaction mixture was treated with 10% trichloroacetic acid and centrifuged (3000 rot/min, 10 min). An aliquot of the upper layer (2.5 mL) was mixed with ultrapure water and 0.1% ferric chloride (2.5 and 0.5 mL, respectively). The absorbance at 700 nm was recorded after 90 s. Higher absorbance values indicate stronger reducing activity [26,80,81].

### 4.6. Cytotoxic Activity on HeLa Cells

#### 4.6.1. Cell Culture

For the experiments, stabilized cultures of human cervical cancer cells (HeLa, ATCC^®^ CCL2^TM^), uncontaminated with *Mycoplasma* sp., were used. HeLa cells were grown in DMEM containing fetal bovine serum (10%), streptomycin (100 µg/mL), penicillin (100 UI/mL), and amphotericin B (50 µg/mL) in a humidified incubator (5% CO_2_) at 37 °C [82]. When HeLa cells reached confluence, they were detached from the plate using a solution containing 0.25% trypsin and 0.02% EDTA, centrifuged at 1800 rpm for 2 min (Sigma Sartorius 2–16 PK centrifuge, Gottingen, Germany), and resuspended in DMEM to provide an optimal cell density (1.5 × 10^4^ cells/mL). HeLa cells were further seeded in cell culture wells (0.3 mL/well) and stored in the incubator at 37 °C [83,84,85].

#### 4.6.2. Cell Viability Assay

To assess cell viability, HeLa cells were stained with 7-AAD, a fluorescent dye able to penetrate only the dead cells and intercalate between guanine and cytosine bases of DNA [86]. After 24 h, when the HeLa cell monolayer was formed, the culture medium (0.3 mL) was discarded and replaced with medium containing either the raw bark extract, resveratroloside (**1**), or pinostilbenoside (**2**) (at 25, 50, and 100 µg/mL), or with medium containing the sample solvent (control). After 48 h of treatment, HeLa cells were quickly detached by trypsinization, centrifuged, washed twice with cold PBS, resuspended in the binding buffer (50 µL), and stained with 7-AAD (10 µL/sample). After 30 min cooling on ice in the dark, a volume of 250 µL of the binding buffer was added to each sample; the sample was immediately analyzed by flow cytometry using the blue laser for fluorochrome excitation. The fluorescence was collected using the 670 LP filter (FL3 detector). The flow cytometry data were collected as LMD files and analyzed using Flowing Software (Cell Imaging Core, Turku Centre for Biotechnology, Åbo Akademi University, Turku, Finland).

#### 4.6.3. Apoptosis Assay

To investigate whether the decrease in HeLa cells’ viability was related to apoptosis, a similar protocol was used except that HeLa cells were successively stained with annexin V-FITC (5 µL/sample) and 7-AAD (10 µL/sample). The fluorescence was collected using a 525 BP filter (FL1 detector) for annexin V-FITC-stained HeLa cells and a 670 LP filter (FL3 detector) for 7-AAD-stained HeLa cells [84,87]. In contrast to 7-AAD, annexin V binds to phosphatidylserine expressed on the surface of apoptotic cells. Therefore, annexin V-FITC and 7-AAD staining discriminates viable (annexin V-FITC negative, 7-AAD negative), dead (annexin V-FITC negative, 7-AAD positive), early apoptotic (annexin V-FITC positive, 7-AAD negative), and late apoptotic (annexin V-FITC positive, 7-AAD positive) cells [86]. The raw bark extract, resveratroloside (**1**), and pinostilbenoside (**2**) were tested at 25 and 50 µg/mL. HeLa cells exposed to the sample solvent were used as control. The data were collected and analyzed as described in Section 4.6.2.

#### 4.6.4. Cell Cycle Assay

To investigate the effects on the cell cycle, DNA content in HeLa cells was quantified using NIM-DAPI staining. DAPI, a fluorescent dye, binds to DNA regions rich in adenine and thymine, the fluorescence intensity being proportional to the DNA amount in cells. An increase in the DNA content in a cell cycle phase indicates cell accumulation and phase arrest [83,88]. After 48 h treatment with the raw bark extract or stilbene glycosides (at 25 and 50 µg/mL), HeLa cells were collected by trypsinization, resuspended in DMEM supplemented with 10% fetal bovine serum, and pelleted by centrifugation (1800 rpm, 4 min). The pellets were washed twice with cold PBS, resuspended in NIM-DAPI, and allowed to stain overnight at 4 °C. For the HeLa culture exposed to the sample solvent (control), bark extract, or stilbene glycosides, 20,000 cells were analyzed by flow cytometry, using a 100 W mercury arc lamp, a 355/37 exciter, and a 460 BP filter for fluorescence collection and linear amplification [83]. The data were collected and analyzed as described in Section 4.6.2.

#### 4.6.5. Cell Proliferation Assay

The effects of the raw bark extract and resveratrol derivatives on HeLa cell proliferation were monitored by flow cytometry using staining with a fluorochrome (CFSE). The assay is based on the progressive decrease in fluorescence of CFSE-stained cells as a consequence of successive divisions and equal distribution of CFSE among daughter cells. Higher fluorescence of treated cells compared to the control indicates cell division blockage [89]. HeLa cells were grown in monolayer culture (2 × 10^6^ cells/mL). The cell monolayer was detached by trypsinization, washed twice with cold PBS, centrifuged at 1800 rpm for 4 min, and resuspended in PBS to a density of 1 × 10^6^ cells/mL. CFSE was added to the cell suspension to a final concentration of 1.5 µM with further incubation at 37 °C for 10 min. The staining was blocked by the addition of 100% fetal bovine serum. After another incubation (37 °C, 10 min), HeLa cells were centrifuged (1800 rot/min, 4 min), washed three times with DMEM supplemented with 10% fetal bovine serum, and resuspended in DMEM [84]. The stained HeLa cells were further seeded in 24-well plates (5 × 10^4^ cells/well) and incubated for 24 h followed by 48 h treatment with the raw bark extract or each isolated compound (at 25 and 50 µg/mL). Following the treatment, CFSE-stained HeLa cells were collected by trypsinization and analyzed on a Beckman Coulter Cell Lab Quanta^TM^ SC–MPL flow cytometer (Beckman Coulter, Brea, CA, USA), using a 488 nm (blue) laser (CFSE excitation) and 525 nm bandpass filters (fluorescence collection). The control consisted of HeLa cells treated with the sample solvent. The data were collected and analyzed as described in Section 4.6.2.

### 4.7. Statistical Analysis

Antioxidant assays were performed in triplicate, and the results were expressed as mean ± standard deviation (SD). HeLa cell-based assays were performed in triplicate; the results were expressed as mean ± standard error (SE). The differences between the results were tested using one-way ANOVA with Tukey’s HSD test (SPSS version 18.0); *p* < 0.05 was considered statistically significant.

## 5. Conclusions

In this study, resveratroloside (**1**) and pinostilbenoside (**2**) were first isolated from *Pinus cembra* L. bark. This is the first report of these compounds in this species. Their structures were confirmed by ^1^H-NMR, ^13^C-NMR, and HRESIMS. Compared to the raw bark extract, resveratroloside (**1**) and pinostilbenoside (**2**) showed lower activity as free radical scavengers and reducing agents. However, they were more effective in reducing the viability and suppressing the proliferation of human cervical carcinoma HeLa cells. At 25 µg/mL, both compounds induced S-phase cell cycle arrest in HeLa cells. At 25 and 50 µg/mL, they significantly reduced the viability of HeLa cells, mainly through non-apoptotic mechanisms. Glycosylated stilbene scaffolds have great potential for therapeutic applications, so further studies are needed to assess the bioactive potential of resveratroloside (**1**) and pinostilbenoside (**2**).

## Data Availability

The original contributions presented in this study are included in the article/Supplementary Materials. Further inquiries can be directed to the corresponding author.

## Supplementary Materials

The following supporting information can be downloaded at https://www.mdpi.com/article/10.3390/plants14101459/s1. Figure S1: ^1^H NMR spectra of resveratroloside (**1**) (A) and pinostilbenoside (**2**) (B). Figure S2: ^13^C NMR spectra of resveratroloside (**1**) (A) and pinostilbenoside (**2**) (B). Figure S3: HRESIMS spectra of resveratroloside (**1**) (A) and pinostilbenoside (**2**) (B).
