# Supplementary material for: Stilbene Glycosides in Pinus cembra L. Bark: Isolation, Characterization, and Assessment of Antioxidant Potential and Antitumor Activity on HeLa Cells
Source: Plants (Basel). 2025 May 14;14(10):1459. doi: 10.3390/plants14101459 (PMC12115102; doi:10.3390/plants14101459)
Supplement: Supplementary file 1 [file plants-14-01459-s001.zip › plants-3622260-supplementary.pdf]

A.

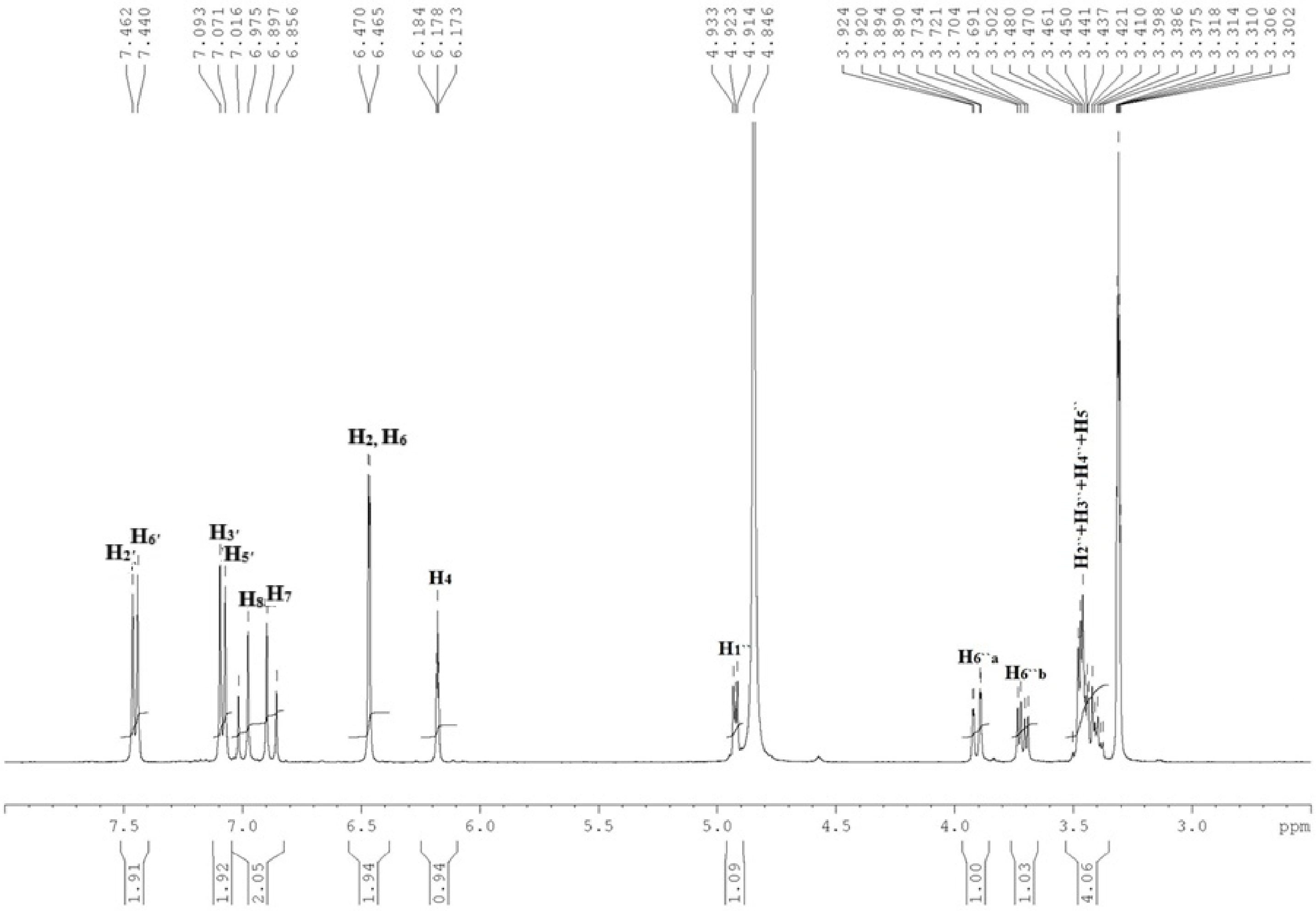
7.462
7.440
7.093
7.071
7.016
6.975
6.897
6.856
6.470
6.465
6.184
6.178
6.173
4.933
4.923
4.914
4.846
3.924
3.920
3.894
3.890
3.734
3.721
3.704
3.691
3.502
3.480
3.470
3.461
3.450
3.441
3.437
3.421
3.410
3.398
3.386
3.375
3.318
3.314
3.310
3.306
3.302
H2′
H6′
H3′
H5′
H8
H7
H2, H6
H4
H1''
H6''a
H6''b
H2''+H3''+H4''+H5''
7.5
7.0
6.5
6.0
5.5
5.0
4.5
4.0
3.5
3.0
ppm
1.91
1.92
2.05
1.94
0.94
1.09
1.00
1.03
4.06

B.

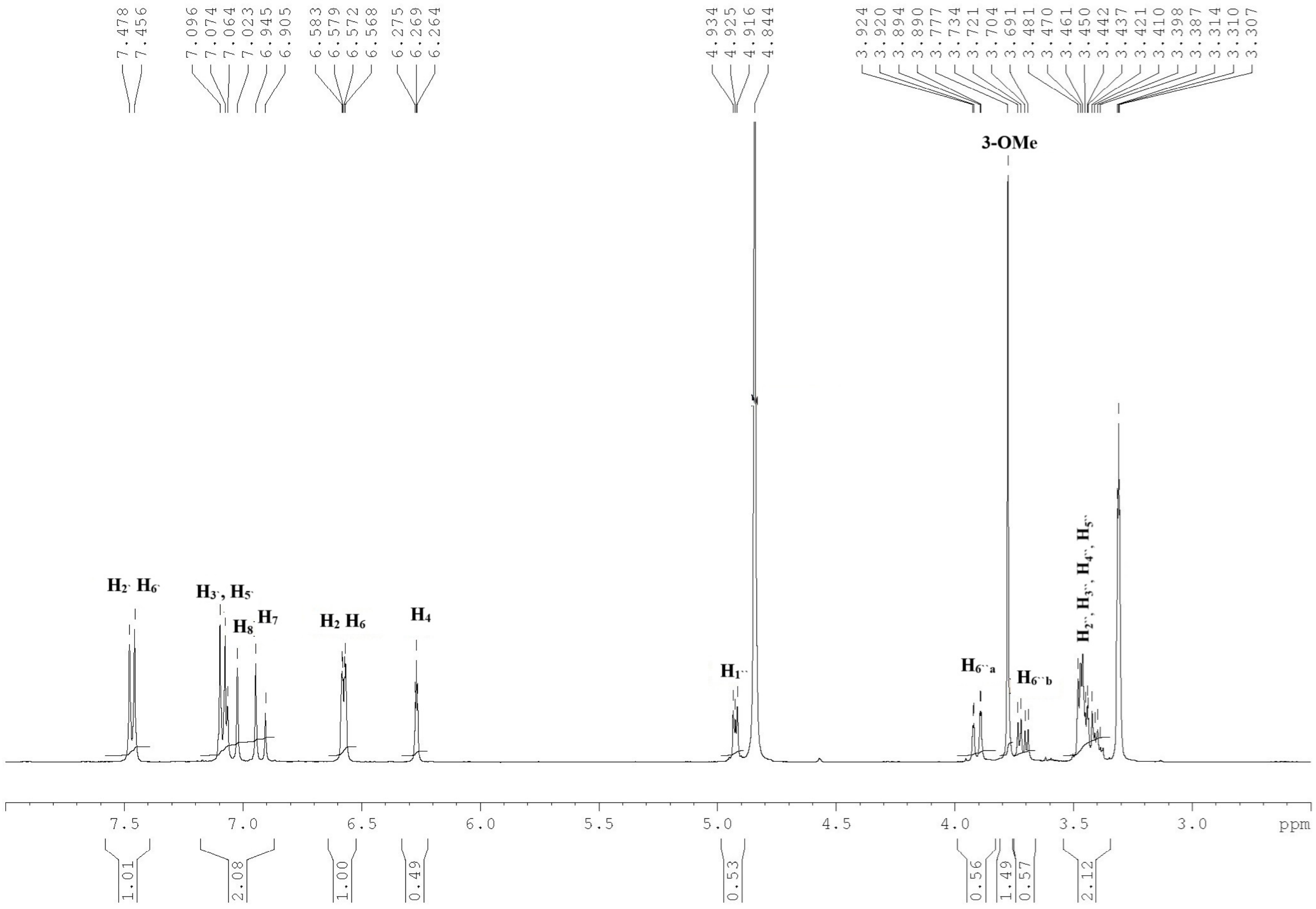


**Figure S1.** $^1$H NMR spectra of resveratroloside (**1**) (A) and pinostilbenoside (**2**) (B).

A.

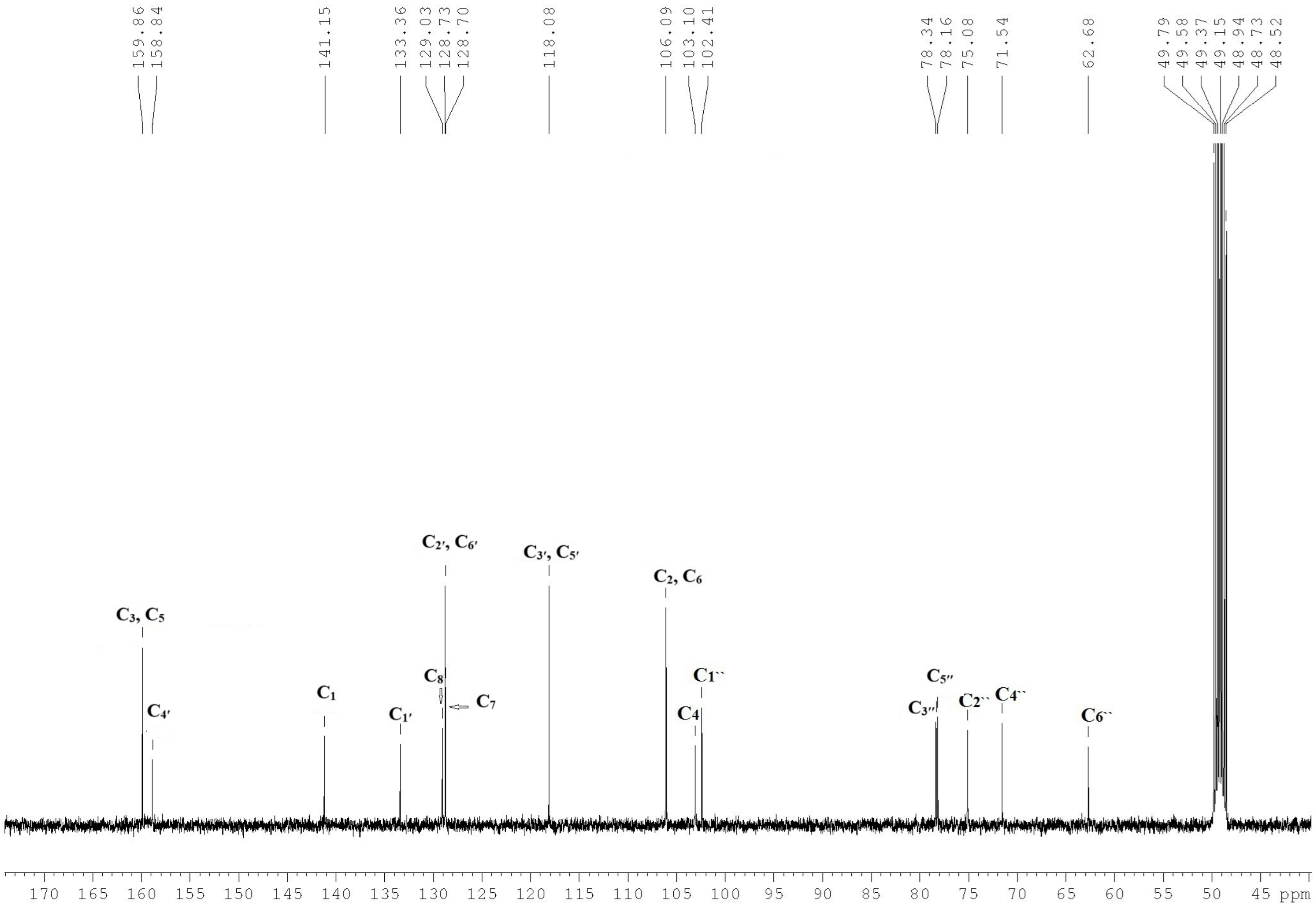
159.86
158.84
141.15
133.36
129.03
128.73
128.70
118.08
106.09
103.10
102.41
78.34
78.16
75.08
71.54
62.68
49.79
49.58
49.37
49.15
48.94
48.73
48.52
C3, C5
C4′
C1
C1′
C8
C7
C2′, C6′
C3′, C5′
C2, C6
C4
C1``
C3′′
C5′′
C2``
C4``
C6``
170 165 160 155 150 145 140 135 130 125 120 115 110 105 100 95 90 85 80 75 70 65 60 55 50 45 ppm

B.

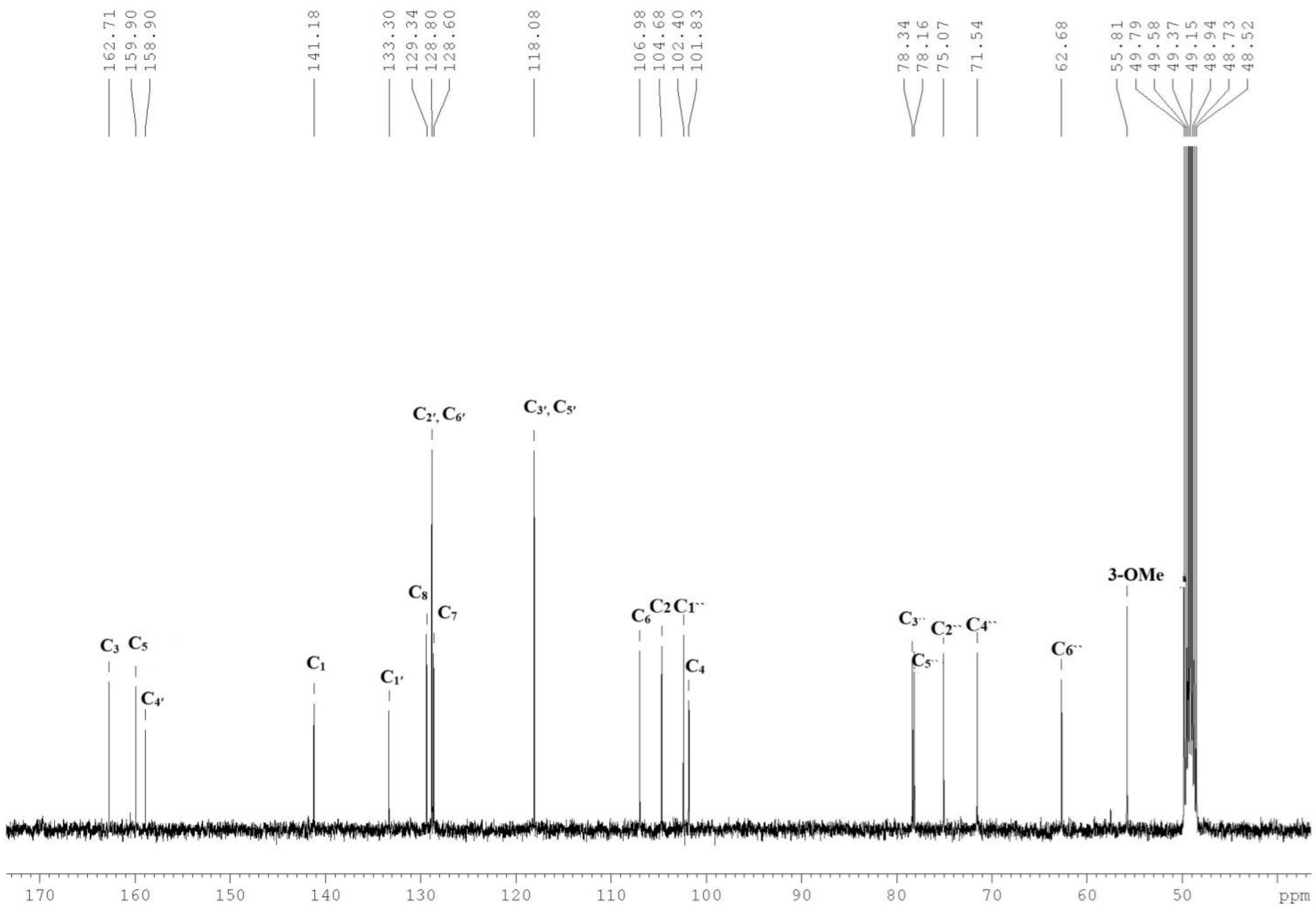


**Figure S2.** $^{13}C$ NMR spectra of resveratroloside (**1**) (A) and pinostilbenoside (**2**) (B).

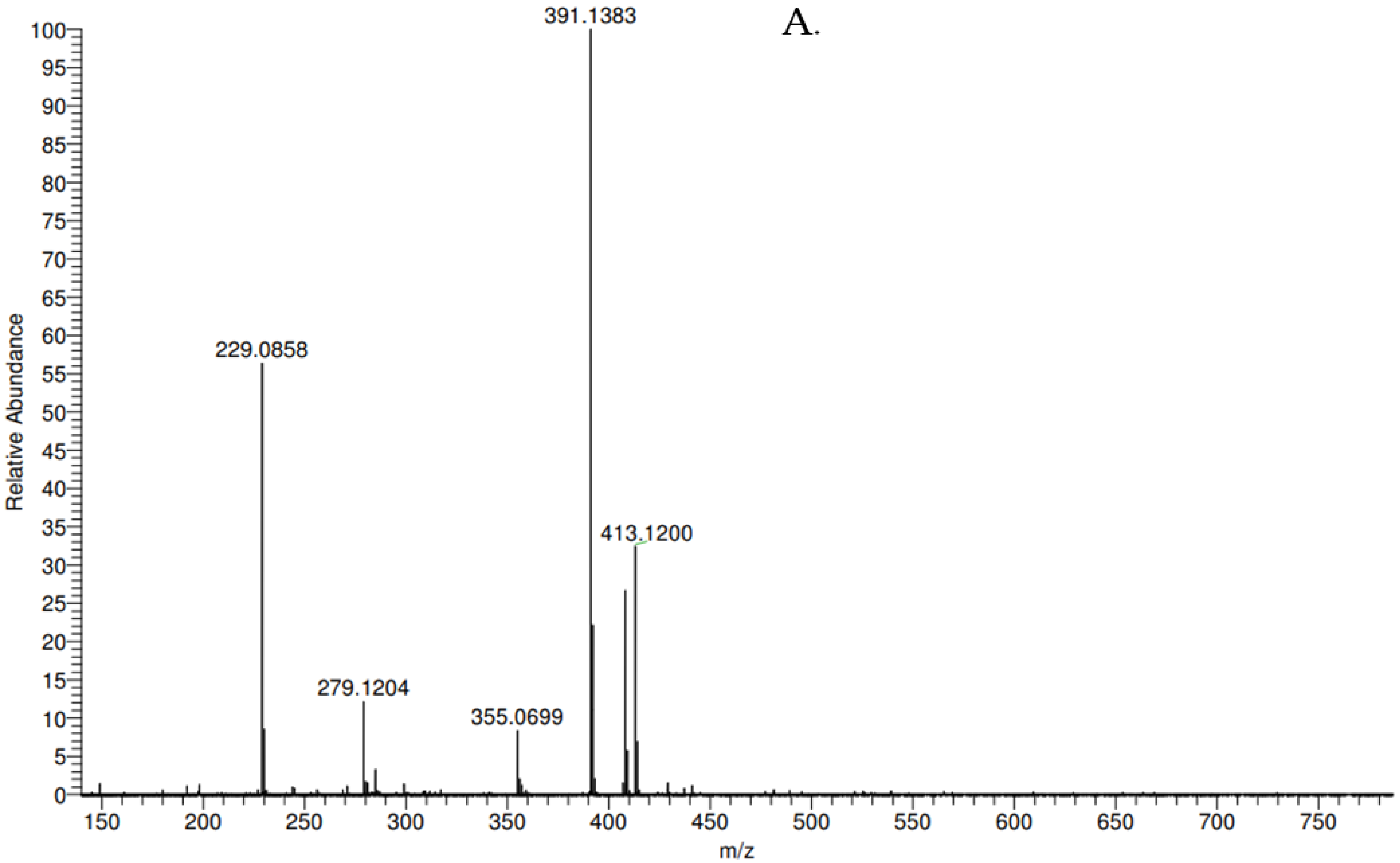

A.
391.1383
229.0858
279.1204
355.0699
413.1200
Relative Abundance
m/z

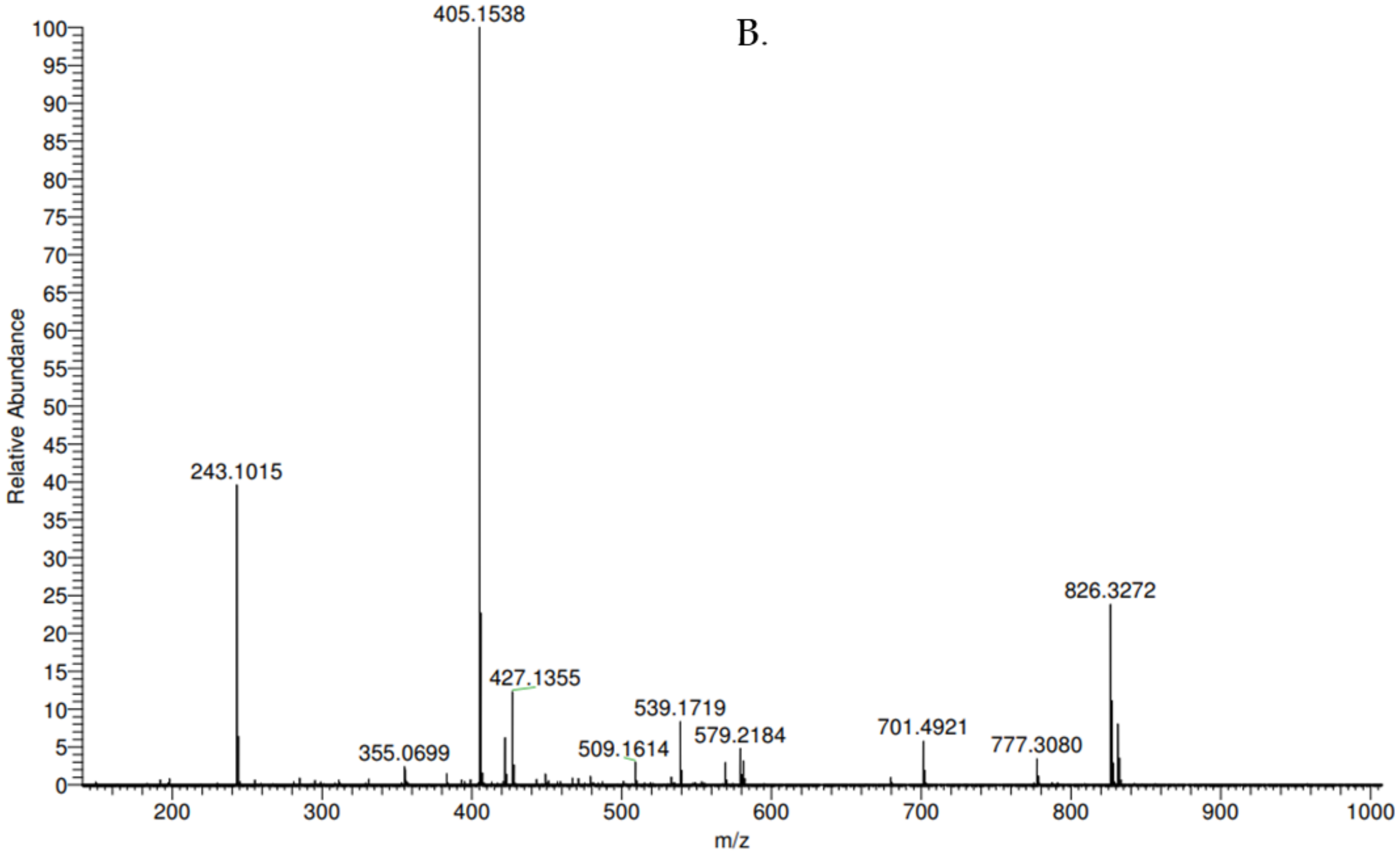


**Figure S3.** HRESIMS spectra of resveratroloside (**1**) (A) and pinostilbenoside (**2**) (B).
